# Intracameral antibiotic prophylaxis and surgical expertise: key determinants in endophthalmitis after cataract surgery

**DOI:** 10.1186/s40942-026-00845-y

**Published:** 2026-04-07

**Authors:** Vinicius Campos Bergamo, Luis Filipe Nakayama, Nilva Simeren Bueno de Moraes, Caio Vinicius Saito Regatieri, Ivan Maynart Tavares, Mauro Silveira de Queiroz Campos, Ana Luisa Hofling-Lima, Maurício Maia

**Affiliations:** 1 Present Address: Retina Division, Department of Ophthalmology, Escola Paulista de Medicina, Hospital São Paulo, Universidade Federal de São Paulo, São Paulo, Brazil; 2 Glaucoma Division, Department of Ophthalmology, Escola Paulista de Medicina, Hospital São Paulo, Universidade Federal de São Paulo, São Paulo, Brazil; 3 Laboratory of Ocular Microbiology, Escola Paulista de Medicina, Hospital São Paulo, Universidade Federal de São Paulo, São Paulo, Brazil; 4 Cornea and External Diseases Division, Escola Paulista de Medicina, Hospital São Paulo, Universidade Federal de São Paulo, São Paulo, Brazil; 5 Laboratory for Computational Physiology, Institute for Medical Engineering and Science, Massachusetts Institute of Technology, Cambridge, MA USA

**Keywords:** Anti-VEGF, Endophthalmitis, Intravitreal injections, Retina, Vitrectomy

## Abstract

**Background:**

Postoperative endophthalmitis is a rare but severe complication of cataract surgery. Intracameral moxifloxacin prophylaxis has been widely adopted, but concerns remain regarding bacterial resistance and clinical outcomes. This study aimed to evaluate the impact of intracameral moxifloxacin prophylaxis on endophthalmitis incidence, microbiological patterns, antibiotic resistance, and visual outcomes in a university hospital setting, with comparison to a private hospital cohort.

**Methods:**

This retrospective cohort study analyzed 21,178 cataract surgeries performed at a university hospital (2014–2023). The incidence of endophthalmitis, microbiological profiles, resistance patterns, and visual acuity outcomes (LogMAR) were assessed. Intracameral moxifloxacin prophylaxis was introduced in 2019. A comparative analysis was conducted with 19,360 surgeries from a private hospital. Fisher’s exact test, Mann-Whitney U test, and Joinpoint regression were used for statistical analysis.

**Results:**

Endophthalmitis incidence at the university hospital was 0.109% (23/21,178 surgeries), significantly higher than the 0.021% incidence in the private hospital (*p* = 0.002). Post-prophylaxis, infection rates declined from 0.219% in 2016 to 0.042% in 2023 (*p* = 0.003). *Staphylococcus epidermidis* predominated (52.6%), and moxifloxacin resistance remained stable (pre: 45.5%; post: no increase).

**Conclusions:**

Intracameral moxifloxacin significantly reduced endophthalmitis incidence without increasing bacterial resistance. Post-prophylaxis infection rates aligned with national surveillance benchmarks (e.g., SES-SP threshold of 0.07%), reinforcing the efficacy of this preventive strategy in a public university setting. The worsening of final visual acuity post-prophylaxis underscores the need for continued clinical vigilance. Ongoing microbiological surveillance remains essential.

## Introduction

Postoperative endophthalmitis is a rare but severe intraocular infection that remains one of the most feared complications following cataract surgery, with potential for profound visual impairment and irreversible blindness [1]. Despite its low incidence, ranging from 0.04% to 0.3% [2–7], its devastating consequences demand strict preventive strategies and prompt intervention.

Historically, improvements in surgical techniques, surgical equipment and prophylactic protocols have contributed to a gradual decline in infection rates, yet the condition continues to pose a clinical challenge, particularly in light of emerging antimicrobial resistance and shifts in microbiological profiles [8, 9].

Multiple patient-related and surgical factors influence the risk of postoperative endophthalmitis. Advanced age, diabetes mellitus, prolonged operative time, posterior capsule rupture, and poor intraoperative wound integrity have been identified as key risk factors [10–12]. 

Intracameral antibiotic prophylaxis, particularly with moxifloxacin, has gained acceptance globally due to its demonstrated efficacy in reducing infection rates [13–15]. Following international trends, Federal University São Paulo - Hospital São Paulo - Ophthalmology Department in Brazil introduced routine intracameral moxifloxacin (Vigamox^®^, Alcon Laboratories) prophylaxis in January 2019.

Prior to this date, no routine intracameral antibiotic prophylaxis was performed at our institution. Moxifloxacin was selected over intracameral cefuroxime — recommended by the ESCRS since 2006 — because a commercially available, preservative-free cefuroxime formulation approved for intracameral use (e.g., Aprokam^®^) was not registered in Brazil at the time of protocol implementation, and compounded preparations carry inherent risks of concentration variability and endotoxin contamination. Preservative-free moxifloxacin (Vigamox^®^) offered a commercially reproducible, single-use alternative consistent with practices adopted by other high-volume centers in Brazil and Latin America.

The aim of this study was to retrospectively evaluate the impact of intracameral moxifloxacin prophylaxis on postoperative endophthalmitis incidence, microbiological patterns, bacterial resistance profiles, and visual outcomes in cataract surgeries performed from January 2014 to December 2023. Additionally, a comparative analysis was conducted between the university public hospital and a private facility to assess the influence of institutional factors on infection rates and clinical outcomes.

## Materials and methods

This retrospective cohort analysis included data from all cataract surgeries performed between January 2014 and December 2023 at the Ophthalmology Department of the Federal University of São Paulo (UNIFESP), a Brazilian tertiary university hospital (Hospital São Paulo). Additionally, data from cataract surgeries performed in 2023 at the Ophthalmologic Hospital of Brasília (HOB-OPTY) were also collected. The study was approved by the university’s Ethics Committee (protocol number 0060/2018), and the requirement for informed consent was waived due to its retrospective design.

Clinical, microbiological, antibiotic susceptibility, and visual acuity data (measured in LogMAR) were extracted and analyzed. Data were obtained from the Ocular Microbiology Laboratory of the Department of Ophthalmology, Federal University of São Paulo, as well as from electronic medical records.

Routine prophylactic intracameral administration of preservative-free moxifloxacin (Vigamox^®^, Alcon Laboratories, 5 mg/mL) at a dose of 0.1 mL (0.5 mg) was implemented in January 2019. The drug was used undiluted and injected intracamerally at the conclusion of the surgical procedure, after wound closure and hydration. Postoperatively, all patients received topical moxifloxacin 0.5% (Vigamox^®^) instilled every 4 h for 7 days. Importantly, all other aseptic and antiseptic protocols remained consistent throughout the study period. These included preoperative skin antisepsis with 10% povidone-iodine (PVP-I), instillation of 5% PVP-I in the conjunctival sac, eyelash isolation using sterile drapes and individualized operating rooms.

All antiseptic and aseptic protocols described were uniformly in place since the beginning of the study period in January 2014 and remained unchanged throughout. Cases were excluded if endophthalmitis occurred following procedures other than cataract surgery (including intravitreal injections, glaucoma filtering surgery, corneal transplantation, or vitreoretinal surgery), as well as traumatic and endogenous endophthalmitis. Only eyes with postoperative endophthalmitis occurring within six weeks of cataract surgery with clinical presentation consistent with infectious postoperative endophthalmitis were included.

Microbiological samples were obtained exclusively from confirmed endophthalmitis cases (aqueous and/or vitreous humor collected at the time of diagnosis and treatment). Microorganism identification and antibiotic susceptibility testing were performed at the Ocular Microbiology Laboratory of the Department of Ophthalmology, Federal University of São Paulo (UNIFESP), using automated minimum inhibitory concentration (MIC) determination, following standard laboratory protocols. Statistical analyses included descriptive statistics to summarize categorical and continuous variables. Fisher’s exact test was used to assess associations between categorical variables, while the Mann–Whitney U test was applied to compare non-normally distributed continuous variables. For normally distributed variables, means were compared using Student’s *t*-test. Temporal trends were evaluated using joinpoint regression analysis. A significance level of α = 0.05 was adopted for all statistical tests.

## Results

**Fig. 1:**
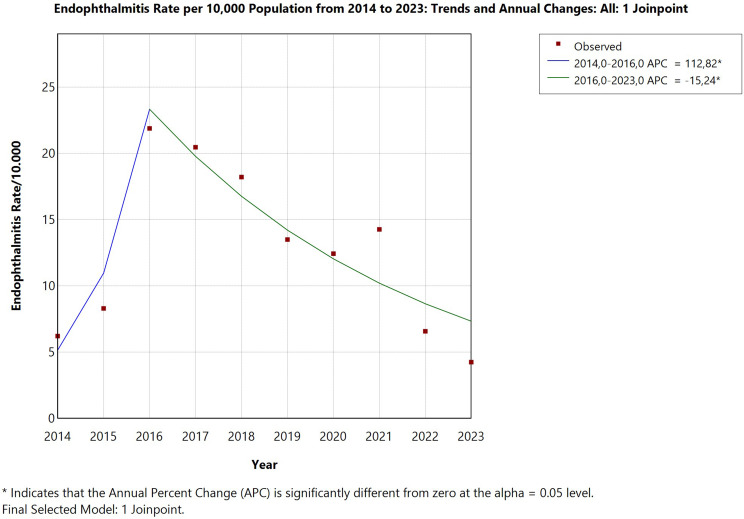
Annual incidence of postoperative endophthalmitis after cataract surgery (2014–2023). A declining trend was observed following the introduction of intracameral moxifloxacin in 2019

**Fig. 2:**
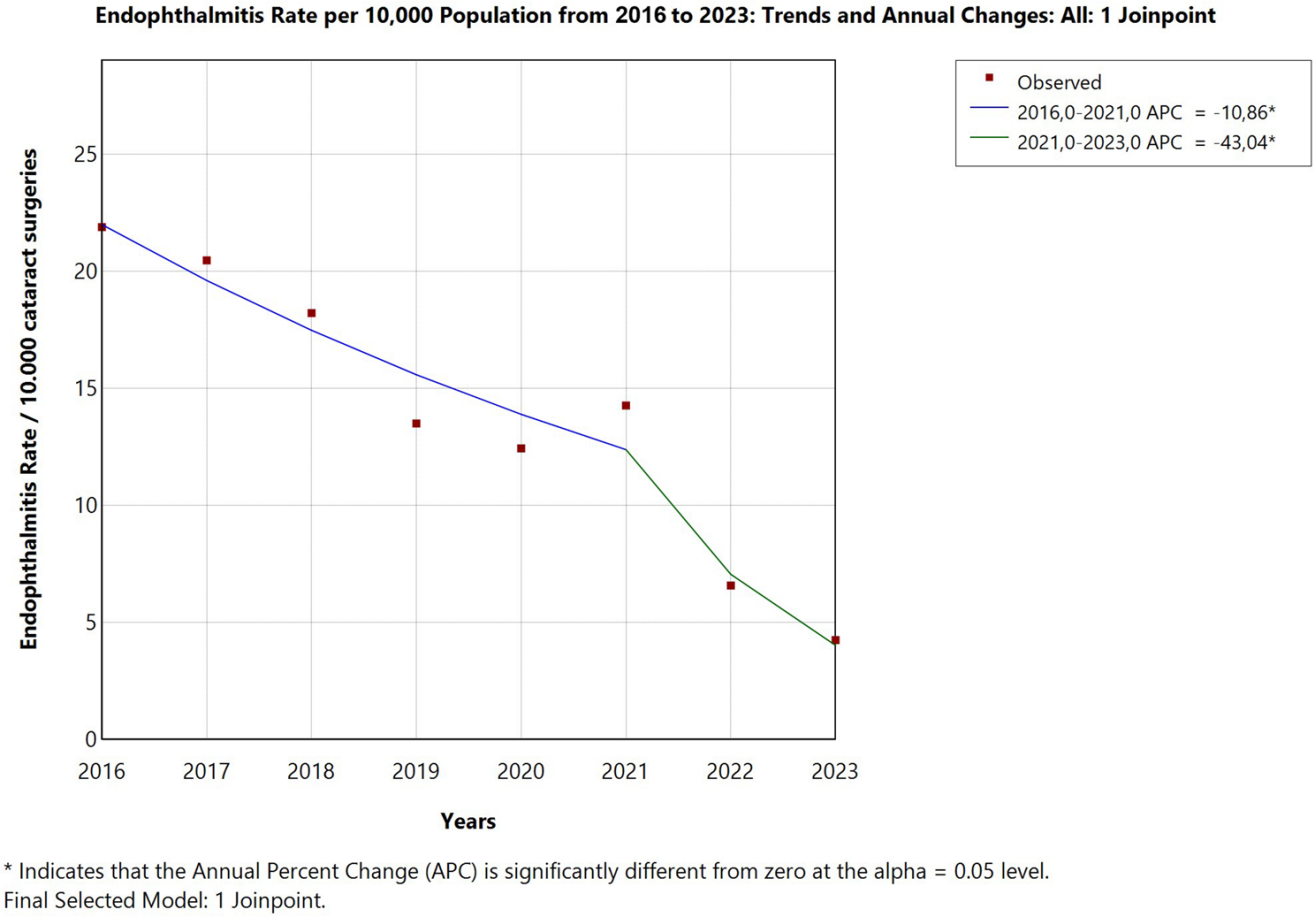
Joinpoint regression analysis of endophthalmitis incidence (2014–2023). A significant annual reduction of − 15.81% (95% CI: − 24.94 to − 5.58; p = 0.003) was observed after prophylaxis implementation

Between January 2014 and December 2023, a total of 21,178 cataract surgeries were performed at the university hospital, with 23 confirmed cases of postoperative endophthalmitis, corresponding to an overall incidence of 0.109% (1.09 cases per 1,000 surgeries). Before the introduction of intracameral moxifloxacin prophylaxis in 2019, annual incidence rates fluctuated, reaching peaks of 0.219% in 2016 and 0.205% in 2017. Following prophylaxis implementation, a progressive decline was observed, with infection rates dropping to 0.066% in 2022 and 0.042% in 2023. Joinpoint regression analysis identified a statistically significant downward trend over the full study period (Annual Percentage Change: -15.81%; 95% CI: -24.94 to -5.58; *p* = 0.003), though this result should be interpreted with caution given the small annual event counts. To complement this analysis, a direct comparison of pre-prophylaxis (2014–2018) vs. post-prophylaxis (2019–2023) incidence rates was performed using Fisher’s exact test, confirming a statistically significant reduction in endophthalmitis incidence following protocol implementation (*p* = 0.034). (Figures 1 and 2).

Table 1 presents the annual distribution of endophthalmitis cases, including the total number of surgeries performed and the corresponding incidence rates for each year.

**Table 1:** Annual incidence of postoperative endophthalmitis following cataract surgery (2014–2023)

Year	Total Surgeries	Endophthalmitis Cases	Positive Cultures	Endophthalmitis Rate (%)
2014	3221	1	1	0,031%
2015	2411	1	1	0,041%
2016	2284	5	4	0,219%
2017	1954	4	3	0,205%
2018	2196	3	2	0,137%
2019	2222	3	1	0,135%
2020	1609	2	1	0,124%
2021	1402	2	1	0,143%
2022	1522	1	1	0,066%
2023	2357	1	1	0,042%
	**21,178**	**23**	**16**	**0**,**109%**

Statistical analyses, including Fisher’s exact test and independent Student’s t-test, demonstrated no significant differences in baseline patient characteristics between the pre- and post-prophylaxis groups, including age, diabetes status, and history of prior surgeries. These findings are summarized in Table 2.

**Table 2:** Comparison of baseline characteristics between pre- and post-prophylaxis groups (2014–2018 vs. 2019–2023)

	Years		*p*
	2014 to 2018	2019 to 2023	
Age (years), N Mean ± SD	14 66.64 ± 13.88	9 63.11 ± 19.85	0.825^a^
VA, N Mean ± SD	4 1.59 ± 0.98	9 1.15 ± 1.13	0.310^a^
Culture, n(%)			0.363^b^
No	3/14 (21.4)	4/9 (44.4)	
Yes	11/14 (78.6)	5/9 (55.6)	
PCR, n(%)			0.657^b^
No	10/14 (71.4)	5/9 (55.6)	
Yes	4/14 (28.6)	4/9 (44.4)	
Previous/Combined Glaucoma Surgery, n(%)			1.000^b^
No	12/14 (85.7)	8/9 (88.9)	
Yes	2/14 (14.3)	1/9 (11.1)	
Diabetes, n(%)			1.000^b^
No	10/14 (71.4)	6/9 (66.7)	
Yes	4/14 (28.6)	3/9 (33.3)	

p - descriptive level of the Mann-Whitney test (^a^) and Fisher’s Exact test (^b^)

VA – Visual Acuity; PCR – Posterior Capsule Rupture

Throughout the study period, Gram-positive bacteria predominated, particularly *Staphylococcus epidermidis* and coagulase-negative staphylococci, which together accounted for 52.6% of all positive cultures. An increase in bacterial diversity was observed post-prophylaxis, with isolated cases of Gram-negative infections, including *Pseudomonas aeruginosa*. These findings are summarized in Table 3.

**Table 3:** Microbiological profile of endophthalmitis cases before and after intracameral moxifloxacin prophylaxis (2014–2018 vs. 2019–2023)

Pre-prophylaxis (2014-18)				Post-prophylaxis (2019-23)		
S. epidermidis	4	36.36%		S. coagulase negative	2	28.57%
S. coagulase negative	2	18.18%		S. aureus	2	28.57%
S. aureus	2	18.18%		Streptococcus orali (viridans group)	1	14.29%
Strepto pneumoniae	1	9.09%		Micrococcus luteus	1	14.29%
Morganella morganii	1	9.09%		Pseudomonas aeruginosa	1	14.29%
Anaerobium Gram negative bacillus	1	9.09%			**7**	**100%**
	**11**	**100%**				

Regarding antibiotic resistance patterns, prior to prophylaxis implementation (2014–2018), moxifloxacin resistance was detected in 45.5% of isolates, while 66.6% of strains were resistant to oxacillin, and moderate to high resistance to cephalosporins was observed. Vancomycin remained fully effective (100% sensitivity) throughout the study period. In the post-prophylaxis period (2019–2023), no statistically significant increase in moxifloxacin resistance was detected. Vancomycin retained its efficacy, and while a slight increase in Gram-negative isolates was noted, there was no clear evidence of a shift in overall pathogen virulence. These findings are summarized in Table 4.

**Table 4:**
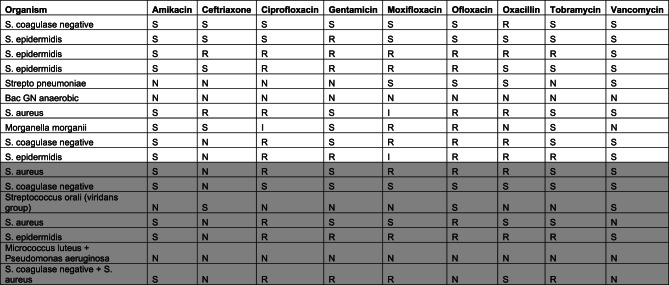
Antibiotic susceptibility patterns of isolated microorganisms in endophthalmitis Cases (Pre- and Post-Prophylaxis Periods)

Post-Prophylaxis period is highlighted

Final visual acuity (VA) outcomes varied among patients, with LogMAR values distributed across a broad range. Following the introduction of intracameral moxifloxacin prophylaxis, there was no statistically significant difference in VA between pre- and post-prophylaxis groups (Mann-Whitney U test, *p* = 0.670). Severe visual impairment (LogMAR ≥ 2.8) was observed in a subset of cases in both the pre- and post-prophylaxis periods and is not attributable to the prophylaxis itself. These findings indicate that while prophylaxis significantly reduced the overall incidence of endophthalmitis, breakthrough infections — likely caused by more virulent organisms or presenting with delayed diagnosis — can still result in poor visual outcomes regardless of prophylactic strategy.

A comparative analysis was conducted using data from a private hospital, where 19,360 cataract surgeries were performed within the year of 2023. Only four cases of postoperative endophthalmitis were recorded in this setting, resulting in a significantly lower incidence rate of 0.021%.

Importantly, the private hospital also employed the same intracameral prophylactic protocol as our institution: preservative-free moxifloxacin (Vigamox^®^, Alcon Laboratories, 0.1 mL) administered at the conclusion of each surgical procedure.

Regarding time to diagnosis, endophthalmitis was identified at a median of 5 to 7 days following cataract surgery across all confirmed cases, consistent with the typical presentation window for acute-onset postoperative endophthalmitis. This interval did not differ significantly between the pre- and post-prophylaxis periods.

Culture positivity rates declined following the introduction of intracameral prophylaxis. Before 2019, the average culture positivity rate was 81.25%, indicating that most infections were microbiologically confirmed. However, after the introduction of prophylaxis, positivity rates declined to 62.5%, suggesting a reduced bacterial load at the time of infection or an increase in culture-negative cases. This reduction may indicate that intracameral prophylaxis effectively lowered bacterial inoculum, leading to milder infections or more challenging microbiological detection.

## Discussion

This study provides strong evidence that routine intracameral moxifloxacin prophylaxis significantly reduces the incidence of postoperative endophthalmitis following cataract surgery.

In 2023, our institution achieved a postoperative endophthalmitis rate of 0.042%, which compares favorably with the 0.06% incidence observed across 81,152 cataract surgeries monitored by the São Paulo State Epidemiological Surveillance System for Endophthalmitis (SIVEN) between 2017 and 2019 [16]. Although the Brazilian Health Regulatory Agency (ANVISA) does not define a strict national benchmark, it mandates monthly reporting and monitoring of endophthalmitis cases as part of its national infection surveillance system. Our findings meet these surveillance goals, underscoring both the effectiveness of intracameral prophylaxis and institutional adherence to national standards.

The implementation of intracameral moxifloxacin marked a significant shift in infection control practices, addressing concerns over infection rates observed in previous years. While variability in rates was evident before its adoption, the introduction of this prophylactic measure coincided with a notable downward trend, reinforcing the efficacy of intracameral antibiotic prophylaxis in mitigating postoperative infections.

The observed reduction in endophthalmitis incidence aligns with previous studies, particularly the landmark trial by the European Society of Cataract and Refractive Surgeons (ESCRS), which demonstrated a fourfold decrease in infection rates with intracameral cefuroxime [17, 18]. Similar findings have been reported with moxifloxacin, especially in high-volume surgical centers [13, 14, 19]. Our results further validate its effectiveness in a university-based public hospital setting, where the baseline infection risk may be inherently higher due to factors such as increased surgical volume, resident participation, and variations in aseptic technique [20, 21]. 

Consistent with previous literature, microbiological analysis confirmed that Gram-positive bacteria, particularly *Staphylococcus epidermidis* and coagulase-negative staphylococci, were the predominant pathogens in both pre- and post-prophylaxis periods [22–24]. The introduction of intracameral moxifloxacin did not significantly alter the microbiological profile, although a slight increase in Gram-negative isolates, including *Pseudomonas aeruginosa*, was noted post-prophylaxis.

Concerns have been raised regarding the potential for intracameral antibiotic prophylaxis to induce bacterial resistance, particularly with widespread fluoroquinolone use [25, 26]. However, our findings indicate that this risk has not yet materialized. Pre-prophylaxis, moxifloxacin resistance was detected in 45.5% of isolates, with significant resistance also noted for oxacillin (66.6%) and cephalosporins. In the post-prophylaxis period, no statistically significant increase in moxifloxacin resistance was observed, and vancomycin retained 100% efficacy.

Although the 45.5% pre-prophylaxis moxifloxacin resistance rate among culture-positive isolates may raise the question of whether intracameral vancomycin would be a superior prophylactic choice, this figure reflects resistance among a selected, virulent subset of pathogens and does not represent the susceptibility profile of the broader periocular flora [27]. More importantly, intracameral vancomycin has been associated with hemorrhagic occlusive retinal vasculitis (HORV), a rare but potentially devastating complication causing severe, irreversible visual loss [28, 29]. This risk has led the American Academy of Ophthalmology and international guidelines to advise against routine prophylactic intracameral vancomycin use [30]. Preservative-free moxifloxacin therefore remains the most appropriate available option in the Brazilian context [31, 32]. 

These findings suggest that intracameral moxifloxacin remains an effective prophylactic agent, with no significant increase in bacterial resistance observed during the study period. Despite theoretical concerns regarding the widespread use of intracameral fluoroquinolones, continued microbiological surveillance is essential to detect potential resistance shifts over time.

Although there appeared to be a clinical trend towards improved final visual acuity outcomes following the introduction of intracameral moxifloxacin prophylaxis, statistical analysis (Mann-Whitney U test, *p* = 0.670) showed no significant difference between pre- and post-prophylaxis groups, consistent with previous literature [33]. This finding suggests that while intracameral moxifloxacin effectively reduced overall endophthalmitis incidence, its impact on the severity of individual infections, as measured by final visual acuity, was less clear.

A possible explanation for the lack of significant improvement in final visual acuity despite the reduction in overall incidence is that intracameral prophylaxis may preferentially prevent low-inoculum or less virulent infections, while breakthrough cases tend to represent biologically more aggressive pathogens or delayed clinical presentations. In this context, prophylaxis shifts the epidemiological profile of endophthalmitis rather than eliminating its most severe forms. Similar patterns have been described in prior epidemiological studies, in which declining incidence did not necessarily correlate with improved visual prognosis among residual cases [11]. As incidence declines, the remaining cases may become disproportionately enriched with infections caused by highly virulent organisms, antibiotic-resistant strains, or host-related vulnerability factors, all of which are associated with poorer visual outcomes [12]. 

This epidemiological shift carries important clinical implications. A lower baseline incidence may paradoxically reduce clinical suspicion, potentially contributing to delayed recognition in rare breakthrough cases. When endophthalmitis becomes less frequent, both physicians and patients may become less alert to early symptoms, which can postpone diagnosis and treatment. Delayed presentation has consistently been identified as a major determinant of poor visual prognosis in postoperative endophthalmitis [3, 22]. These findings reinforce the need for sustained postoperative vigilance, strict follow-up protocols, and continuous education of healthcare providers and patients, ensuring that declining incidence does not translate into complacency in clinical practice.

Another potential factor influencing visual acuity outcomes relates to the timing and aggressiveness of management. Delayed intervention or initial underestimation of infection severity may contribute to suboptimal visual results in a subset of patients. Classic and contemporary studies have demonstrated that early vitrectomy and rapid therapeutic escalation can improve outcomes in selected cases of severe endophthalmitis [34]. Future research should further clarify which breakthrough infections benefit from early surgical intervention, particularly in the era of widespread intracameral prophylaxis.

A noticeable finding in this study was the lower infection rate observed in the private hospital cohort compared to the university hospital (0.021% vs. 0.042% in our best year, 2023), despite both institutions using identical intracameral prophylactic protocols (preservative-free moxifloxacin, Vigamox^®^, 0.1 mL). This comparison should be interpreted as hypothesis-generating, given the differences in time frames, geographic location, and case mix between the two cohorts. Nevertheless, this finding suggests that factors beyond pharmacological prophylaxis — including surgical expertise, institutional infection control measures, and surgical training environment — may be independent determinants of postoperative infection risk. At the university hospital, cataract surgeries were performed by both attending ophthalmologists and residents in training, whereas in the private hospital, all procedures were conducted by fully trained surgeons. Although individual surgeon-level data were not uniformly available in our retrospective dataset and a direct attribution could not be established, prior literature has consistently associated trainee-performed cataract surgery with higher complication rates — likely due to prolonged surgical times, increased intraocular manipulation, and greater variability in technique — and this represents a plausible contributor to the observed institutional difference [20]. Additionally, high-volume university hospitals, particularly those publicly funded, often face resource constraints and higher patient turnover, potentially affecting surgical duration, surgical efficiency, and postoperative surveillance. In contrast, private hospitals may benefit from more controlled surgical environments, stricter operating room protocols, more standardized surgical practices, and enhanced postoperative monitoring, all of which could contribute to lower infection rates.

The relevance of intracameral antibiotic prophylaxis in reducing postoperative endophthalmitis has also been demonstrated in other public healthcare settings. A recent Brazilian study by Salha et al. [35] evaluated the introduction of intracameral cefuroxime in a tertiary public hospital where cataract surgeries were predominantly performed by residents. Following implementation, the incidence of endophthalmitis dropped from 0.27% to 0%, with no reported adverse effects​. These findings emphasize not only the effectiveness and safety of intracameral prophylaxis, but also its critical role in public teaching hospitals, where inherent challenges such as variability in surgical expertise and limited resources may increase infection risk.

Beyond institutional differences, socioeconomic disparities may also play a role in postoperative infection risk. Patients from lower socioeconomic backgrounds may face barriers to accessing high-quality healthcare services, including delayed surgical care, limited access to postoperative follow-up, and difficulty in adhering to medical recommendations. Studies have suggested that poorer living conditions, reduced health literacy, and financial constraints may contribute to suboptimal perioperative hygiene and lower compliance with postoperative care protocols, potentially increasing susceptibility to infectious complications, including endophthalmitis [21]. Additionally, patients treated in public hospitals often experience longer wait times for surgery and a higher burden of systemic comorbidities, such as diabetes, which has been associated with an increased risk of postoperative infections [1, 10, 11, 36]. 

Although this study did not directly assess the impact of socioeconomic and institutional factors, the significant difference in infection rates between the public university hospital and the private hospital highlights the need for further investigation into how socioeconomic determinants, surgical training programs, and institutional infection control policies influence postoperative infection risk and visual outcomes.

These findings reinforce the efficacy of intracameral moxifloxacin in reducing infection rates but emphasize the necessity of maintaining a high index of suspicion for endophthalmitis. Early recognition, prompt diagnosis, and aggressive management remain crucial, as delays continue to significantly impact visual outcomes [34]. Further research into refining prophylactic and therapeutic strategies, particularly for identifying cases at higher risk of severe outcomes, remains essential.

This study has important clinical implications. Routine intracameral moxifloxacin should remain the standard of care in cataract surgery, given its demonstrated ability to significantly reduce endophthalmitis rates without evident resistance selection. However, the five-fold lower infection rate observed in private hospitals suggests that optimizing infection control strategies in academic settings may further enhance patient safety. Training programs should emphasize surgical asepsis, early infection recognition, and refined intraoperative techniques to minimize infection risks among resident-performed surgeries. Moreover, despite a declining incidence of endophthalmitis, clinicians should remain highly vigilant, as delayed recognition may contribute to worse visual outcomes.

This study has limitations inherent to its retrospective design. Specifically: (1) overall posterior capsule rupture (PCR) rates for the full surgical cohort were not systematically recorded in our institutional database and could therefore not be reported across the study period; while PCR is a recognized risk factor for endophthalmitis, the comparable baseline characteristics between pre- and post-prophylaxis groups argue against this as a major confounding factor; (2) data on treatment modality (tap-and-inject vs. pars plana vitrectomy) were not uniformly available, limiting interpretation of visual acuity outcomes; (3) the Joinpoint regression trend, while statistically significant, should be interpreted with caution given the small annual case numbers; and (4) the inter-institutional comparison is hypothesis-generating — future prospective studies with standardized data collection across multiple centers are needed to confirm these findings. The reduced number of microbiological samples in the post-prophylaxis period limits the ability to detect subtle resistance trends. Furthermore, the differences between hospital settings (private vs. university) may involve confounding variables not accounted for in our analysis. Future prospective, multicenter studies should explore the impact of surgeon experience, institutional infection control measures, and host factors on postoperative endophthalmitis incidence and clinical outcomes.

## Conclusion

In conclusion, routine intracameral moxifloxacin prophylaxis significantly reduced postoperative endophthalmitis incidence without increasing bacterial resistance. However, the observed discrepancy in infection rates between university and private hospitals highlights the critical role of surgical experience and institutional infection control policies. Additionally, it underscores the need for ongoing clinical vigilance, emphasizing early recognition and aggressive management of residual cases. Continued microbiological surveillance is essential to detect potential future resistance trends and to further refine prophylactic strategies in cataract surgery.

## Data Availability

The datasets used and/or analyzed during the current study are available from the corresponding author on reasonable request.
